# Genomic Characterisation of Invasive Non-Typhoidal *Salmonella enterica* Subspecies *enterica* Serovar Bovismorbificans Isolates from Malawi

**DOI:** 10.1371/journal.pntd.0002557

**Published:** 2013-11-14

**Authors:** Christina Bronowski, Maria C. Fookes, Ruth Gilderthorp, Kevin E. Ashelford, Simon R. Harris, Amos Phiri, Neil Hall, Melita A. Gordon, John Wain, Charles A. Hart, Paul Wigley, Nicholas R. Thomson, Craig Winstanley

**Affiliations:** 1 Institute of Infection and Global Health, University of Liverpool, Liverpool, United Kingdom; 2 Pathogen Genomics, The Wellcome Trust Sanger Institute, Hinxton, Cambridge, United Kingdom; 3 Institute of Integrative Biology, University of Liverpool, Liverpool, United Kingdom; 4 Malawi-Liverpool-Wellcome Trust Clinical Research Program, Queen Elizabeth Hospital, Blantyre, Malawi; 5 Department of Medicine, College of Medicine, University of Malawi, Malawi; 6 Department of Medical Microbiology, University of East Anglia, Norwich Research Park, Norwich, United Kingdom; Beijing Institute of Microbiology and Epidemiology, China

## Abstract

**Background:**

Invasive Non-typhoidal *Salmonella* (iNTS) are an important cause of bacteraemia in children and HIV-infected adults in sub-Saharan Africa. Previous research has shown that iNTS strains exhibit a pattern of gene loss that resembles that of host adapted serovars such as *Salmonella* Typhi and Paratyphi A. *Salmonella enterica* serovar Bovismorbificans was a common serovar in Malawi between 1997 and 2004.

**Methodology:**

We sequenced the genomes of 14 Malawian bacteraemia and four veterinary isolates from the UK, to identify genomic variations and signs of host adaptation in the Malawian strains.

**Principal Findings:**

Whole genome phylogeny of invasive and veterinary *S.* Bovismorbificans isolates showed that the isolates are highly related, belonging to the most common international *S.* Bovismorbificans Sequence Type, ST142, in contrast to the findings for *S.* Typhimurium, where a distinct Sequence Type, ST313, is associated with invasive disease in sub-Saharan Africa. Although genome degradation through pseudogene formation was observed in ST142 isolates, there were no clear overlaps with the patterns of gene loss seen in iNTS ST313 isolates previously described from Malawi, and no clear distinction between *S.* Bovismorbificans isolates from Malawi and the UK.

The only defining differences between *S.* Bovismorbificans bacteraemia and veterinary isolates were prophage-related regions and the carriage of a *S.* Bovismorbificans virulence plasmid (pVIRBov).

**Conclusions:**

iNTS *S.* Bovismorbificans isolates, unlike iNTS *S.* Typhiumrium isolates, are only distinguished from those circulating elsewhere by differences in the mobile genome. It is likely that these strains have entered a susceptible population and are able to take advantage of this niche. There are tentative signs of convergent evolution to a more human adapted iNTS variant. Considering its importance in causing disease in this region, *S.* Bovismorbificans may be at the beginning of this process, providing a reference against which to compare changes that may become fixed in future lineages in sub-Saharan Africa.

## Author Summary

Bacteraemia and meningitis caused by non-typhoidal *Salmonella* (including serovars Typhimurium, Enteritidis and Bovismorbificans) are a serious health issue in sub-Saharan Africa, particularly in young children and HIV-infected adults. Previous work has indicated that a distinct *S.* Typhimurium sequence type, ST313, has evolved and spread in these countries, and may be more human-adapted than isolates found in the developed world. We therefore investigated the genomes of *Salmonella enterica* serovar Bovismorbificans bacteraemia isolates from Malawi and compared them to genomes of veterinary *S.* Bovismorbificans isolates from the UK using Next Generation Sequencing Technology and subsequent genomic comparisons to establish if there is a genetic basis for this increase in invasive disease observed among African NTS. Contrary to the previous findings for *S.* Typhimurium, where a distinct ST is found only in sub-Saharan Africa, we discovered that the *S.* Bovismorbificans isolates from Malawi belong to the most common ST of the serovar and the genome is highly conserved across all sequenced isolates. The major differences between UK veterinary and African human isolates were due to prophage regions inserted into the genomes of African isolates, coupled with a higher prevalence of a virulence plasmid compared to the UK isolates.

## Introduction

Invasive Non-typhoidal *Salmonella* (iNTS) are a major cause of morbidity and mortality in sub-Saharan Africa. Especially in young children, iNTS are either the first or second most common cause of bacteraemia [1], [2], meningitis and septic arthritis [3], [4] with high morbidity. HIV infection is the primary risk factor for iNTS bacteraemia in adults, and it has been suggested that iNTS emerged together with the HIV pandemic in sub-Saharan Africa [5]. The most important clinical risk factors for iNTS disease in children are malnutrition, malaria and anaemia, with one in five cases of NTS bacteraemia in children also associated with HIV infection [2], [6], [7]. There is considerable interest in identifying any underlying bacterial genetic basis for the apparent increase in invasiveness and transmission of African NTS strains.

Strain collections of iNTS isolates from Africa are dominated by the serovars Typhimurium and Enteritidis [8]–[12]. However a seven year study of iNTS isolates associated with bacteraemia in Malawi showed that *S.* Bovismorbificans was the third most common serovar, with 46 cases, which accounts for 1% of the total number of NTS isolates [11]. In contrast to this, a study of *Salmonella* bacteraemia in developed countries (Finland, Denmark, Canada and Australia) showed that among 490 bacteraemia NTS isolates, isolated between 2000 and 2007, only one was *S.* Bovismorbificans (0.2%) [13]. However, *S.* Bovismorbificans has been found responsible for gastroenteritis outbreaks: in Malaysia between 1973 and 1996 *S.* Bovismorbificans accounted for 2–11% of salmonellosis cases [14], [15] as well as isolated outbreaks all over Europe and around the world [16], [17] [18], *S.* Bovismorbificans phage type 32 (PT32) [19], [20].

It has been shown that bacteraemia isolates of *S.* Typhimurium from Kenya and Malawi belong to a distinct Multi Locus Sequence Typing (MLST) group, ST313, that harbours a specific repertoire of prophages and shows evidence of specific patterns of genome degradation with many parallels to human-specific *Salmonella* serovars such as *S.* Typhi and Paratyphi A, which cause acute invasive disease [21]. ST313 is significantly distant from the common gastroenteritis-associated *S.* Typhimurium ST19 [22]. These studies have raised the possibility that bacteremia-associated iNTS serovars that were previously able to infect a broad host range are becoming human-host adapted [23], [24].

Multidrug resistance is also a significant factor in the emergence of iNTS strains in Africa, resulting in a reliance on fluoroquinolones [11], [25]. While *S.* Typhimurium and Enteritidis isolates from Malawi exhibited resistance to commonly-used antimicrobials (including ampicillin, co-trimoxazole and chloramphenicol), iNTS *S.* Bovismorbificans isolates have remained comparatively susceptible to these commonly-used antimicrobials.

Here, we report the genome sequence of *S.* Bovismorbificans 3114 (ST142), a paediatric bacteraemia isolate from Malawi, and describe a detailed analysis of iNTS strains of *S.* Bovismorbificans causing disease in humans in Malawi and compare them to other isolates from the same region and from veterinary isolates from the UK. We investigated whether there are markers of adaptation to the human host, similar to those described in other iNTS serovars from this region.

## Materials and Methods

### Bacterial strains and antimicrobial resistance profiling

Malawian *S.* Bovismorbificans isolates used in this study were taken from a previous study by Gordon *et al* [11] and date from 1997 to 2004. *S.* Bovismorbificans serovar designations for Malawian strains were confirmed by serotyping at the National *Salmonella* Reference Laboratory, Galway, Republic of Ireland. Veterinary strains of *S.* Bovismorbificans were obtained from Professor Paul Barrow (University of Nottingham), and were taken from a collection dating from the 1970s and 1980s. All *Salmonella* strains were stored in 10% (v/v) glycerol broth at −80°C. The antimicrobial susceptibility profiles of the four veterinary strains were determined by the disc diffusion method, in accordance with BSAC guidelines (http://bsac.org.uk/wp-content/uploads/2012/02/Version-11.1-2012-Final-.pdf), using a total of 11 antimicrobials: (AML10 (amoxicillin 10 µg), AMC30 (amoxicillin/clavulanic acid 30 µg), CTX30 (cefotaxime 30 µg), CN 10 (gentamicin 10 µg), CIP 1 (ciprofloxacin 1 µg), W 2.5 (trimethoprim 2.5 µg), NA 30 (nalidixic acid 30 µg), RL25 (sulphamethoxazole 25 µg), C10 (chloramphenicol 10 µg), TET30 (tetracycline 30 µg), CXM 5 (cefuroxime sodium 5 µg), RD 2 (rifampicin 2 µg), CAZ30 (ceftazidime 30 µg), S 25 (streptomycin 25 µg). The susceptibility profiles of the human *S.* Bovismorbificans isolates from Malawi have been determined previously, in accordance with BSAC guidelines [11].

### Genomic DNA extraction and genome sequencing

*Salmonella* strains were cultured in Luria broth overnight at 37°C shaking at 200 rpm. Genomic DNA extractions were performed using the Wizard Genomic DNA Purification Kit (A1120, Promega, Madison, USA) as described in the manufacturer's instructions.

The genome of *S.* Bovismorbificans strain 3114 was sequenced using the Roche 454 Genome Sequencer FLX (GS-FLX) following the manufacturer's instructions (Roche 454 Life Science, Branford, CT, USA). In brief, each sample was made into both a paired-end and fragment library using the standard FLX chemistry for 454. Fragment libraries were prepared by fragmentation, attachment of adapter sequences, refinement of the ends and selection of adapted molecules. Paired-end libraries were produced by hydroshear shearing, circularisation, addition of adapters and selection, as for the fragment library. Both libraries were amplified by emPCR and fragment-containing beads were recovered and enriched. Sequencing primers were added and each library was deposited onto a quarter of a PicoTitrePlate plate and sequenced.

Multiplexed Illumina standard libraries were prepared for *S.* Bovismorbificans 3114 and 17 additional strains following standard protocols with 200 bp inserts and sequenced on the Illumina Genome Analyzer II. Paired end sequence runs were performed with 54 bp read length. Raw sequence data is submitted to the public data repository, ENA, under accession ERP000181.

### *S.* Bovismorbificans strain 3114 genome sequence assembly

For *S.* Bovismorbificans 3114 454 data, reads from the fragment and paired-end libraries were de-novo assembled into contigs using the Roche 454 Newbler assembler (version 2.0.01.12) with default settings.

Illumina data was then used to extend and order the 454-assembled contigs using the PAGIT package [26] as follows: 454 contigs were extended using ICORN [26], and the resulting contigs were ordered and orientated with respect to the genome of *S.* Typhimurium LT2 using ABACAS [27]. Finally, gap closure was attempted where possible using IMAGE [26].

For each scaffold-contig in turn, putative coding sequences (CDSs) were predicted using Glimmer version 3.02 (http://www.cbcb.umd.edu/software/glimmer/). A further in-house Perl script was then used to identify and correct those CDSs likely to have been split due to sequencing errors when handling homopolymer repeats. This involved BLASTP alignment of CDS protein translations against a database of translations generated from previously annotated *Salmonella* genomes, the identifications of likely indels within homopolymer regions, the modification of coding sequence feature positions to correct errors, and the merging of relevant CDSs. Where such modification occurred, this was recorded as metadata (in the form of the eventual GenBank feature note field). Such CDSs were also marked with the exception flag set to ‘low-quality sequence region’ for the final GenBank submission to signify poor quality sequencing.

A putative function was then assigned to each gene by BLASTn (NCBI Blast 2.2.17) comparison with a database of sequences generated from the previously annotated genome of *S.* Typhimurium LT2. Putative tRNA genes were detected using tRNAscan-SE 1.23 (ftp://selab.janelia.org/pub/software/tRNAscan-SE/). A pseudochromosome, consisting of a concatenation of contigs arranged into scaffolds, with 100 Ns separating adjoining scaffolds, was prepared for comparison purposes; the final version of the 3114 genome was manually curated using Artemis [28], [29].

The *S.* Bovismorbificans 3114 chromosome has been submitted to EMBL under accession number HF969015, and its 93.8 kb virulence plasmid under accession number HF969016.

### *S.* Bovismorbificans Illumina genome assembly

Illumina sequence data for the 17 additional genomes was assembled as follows: for each strain, Velvet [30] was used to create multiple assemblies by varying the kmer size between 66% and 90% of the read length. From these assemblies, the one with the best N50 was chosen and contigs which were shorter than the insert size length were removed. An assembly improvement step was then run on the chosen assembly. The contigs of the assembly were scaffolded by iteratively running SSPACE [31]. Then gaps identified as 1 or more N's, were targeted for closure by running 120 iterations of GapFiller [32]. Finally, the reads were aligned back to the improved assembly using SMALT (http://www.sanger.ac.uk/resources/software/smalt/) and a set of statistics was produced for assessing the QC of the assembly.

All of the software developed is freely available for download from GitHub (https://github.com/sanger-pathogens) under an open source license, GNU GPL 3. The improvement step of the pipeline is also available as a standalone Perl module from CPAN (http://search.cpan.org/~ajpage/) (see Table S1 for assembly data and statistics).

### Pseudogene analysis

Pseudogenes were identified, using ACT comparisons [33], by comparing the genome of strain 3114, first to *S.* Typhiumurium LT2 and then to the genomes of *S.* Typhimurium D23580, SL1344 and DT104. Pseudogenes were identified according to whether CDS showed frameshift mutations, missing N- or C-terminals or carried nonsense mutations. Once pseudogenes were identified in the 3114 genome, their orthologous sequences were checked in the assemblies of a further six *S.* Bovismorbificans isolates, from both Malawi (3180, D1253, D993, A1668,) and the UK (653308, 276608). These isolates were chosen as representatives of distinct subclades in the tree. In some cases it was not possible to identify mutations due to gaps in the sequence; these are highlighted in Table S5.

### Multi Locus Sequence Typing (MLST)

MLST sequences were obtained from Illumina reads and sequence types were assigned through the MLST website (http://www.mlst.net/). Ambiguous results for some individual loci were subsequently confirmed by PCR amplification of the locus and sequencing of the PCR product (Beckman Coulter). In order to build the MLST-based phylogenetic tree of Figure S1, the concatenated sequences of the seven MLST loci of *S.* Bovismorbificans 3114 and the most common *S. enterica* STs from published databases were loaded into SeaView v3.2 [34]. The phylogeny was reconstructed using PhyML [35] within the Seaview package, and FigTree v1.3.1 [36] was used to edit and label the final figure.

### Construction of a pan genome pseudomolecule

For the purpose of mapping and visualization of the genomic content of all *S.* Bovismorbificans samples, a pseudomolecule was constructed comprising the reference 3114 genome (chromosome and virulence plasmid) and all non-redundant accessory regions found by tblastx in each sample assembly with respect to the others in an iterative manner. Briefly we performed pairwise comparisons, firstly of one isolate against the reference sequence (genome and its plasmid). Regions in the comparator that were not present in the reference were identified and added to the end of the reference sequence to form a pan genome pseudomolecule. This was repeated in an iterative process involving manual curation of the sequences to be included in the growing pseudo molecule.

### Read mapping, single nucleotide polymorphism (SNP) calling and construction of the phylogenetic tree of the *S.* Bovismorbificans sample set

**Figure 1:**
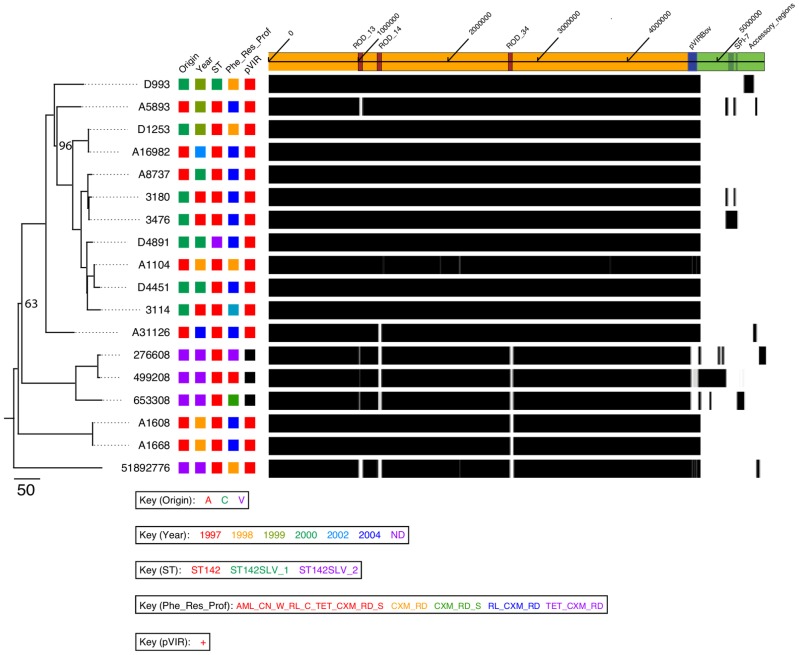
Phylogenetic tree of S. Bovismorbificans isolates and visualization of the Bovismorbificans pangenome. Maximum likelihood phylogenetic tree of *S.* Bovismorbificans isolates (left), Bootstrapping values below 100% are shown and branch length corresponds to SNPs, proportional to the shown scale (left). Colour-coded information on each strain follows to the right, including origin (A = Adult, C = Child, V = Veterinary), year of isolation (an exact date of isolation for the veterinary isolates is not known (ND) but the collection predates the 1980s), ST, antimicrobial resistance profile (RL = sulphamethoxazole, CXM = cefuroxime, RD = rifampicin, amoxicillin (AML), gentamicin (CN), trimethoprim (W), chloramphenicol (C), tetracycline (TET), streptomycin (S)) and presence or absence of the virulence plasimd pVIRBov (see key for further details, bottom). The shaded area (right) shows base positions of the pan-genome pseudomolecule (depicted above) coloured white, grey or black representing 0 (white; absent) 1–14 (grey; partially present) or 15 or more (black; present) read coverage per base for each sample. The pan-genome pseudomolecule is shown (right top) consisting of the chromosome of isolate 3114 genome (ochre shading) the virulence plasmid pVIRBov (blue shading) and concatenated accessory regions (green shading). Significant regions of variation (see methods) are marked on the pan-genome pseudomolecule: RODs 13, 14, 34 (red boxes) and SPI-7 (dark green).

Mapping of illumina reads per sample was carried out against this resulting pseudomolecule using SMALT (http://www.sanger.ac.uk/resources/software/smalt/) without mapping to repeats. SNP calling was performed as previously described [37]. In order to construct a robust phylogenetic tree a Bayesian approach [38], which identifies high density SNPs and recombinant regions and ignores them when constructing the phylogeny, was used. A detailed list of sites removed from the chromosome, 48,423 bases in total, is summarised in Table S2 and the alignment of variant sites used to construct the final phylogenetic tree is presented in Table S3. In order to construct the phylogeentic tree shown in Figure 1 an initial tree using *S.* Heidelberg str SL476 (acc no. CP001120) (see Figure S1), as an outgroup was constructed. This identified the root position in the ingroup and final tree. Using this root position the final tree shown in Figure 1 was constructed using all 954 variant sites (Table S3) within the chomosome: A maximum likelyhood approach (RAxML) was used to construct the initial bipartitions tree followed by a reconstruction of the SNPs onto the tree branches using delayed transformation (DELTRAN) parsimony [39].

Mapped illumina read data was saved in Bam format [40] and converted to coverage files (number of reads mapped to each base coordinate of the reference) with an in-house script. To aid visualisation the read depth per base position of each isolate against the pan-pseudomolecule reference sequence was constructed (Figure 1). Base positions with 0 or 1–14 reads mapped were coloured white or grey, respectively. Base positions with 15× coverage or greater are coloured black. The cutoff of 15 or more times coverage per base position was selected because it was just below the minimum median coverage obtained across all of the isolates we sequenced (16 to 31× coverage [data not shown]; see Figure 1). Using the observed coverage regions >4000 bps showing a significant deviation from the median coverage were identified by manual curation and checked against the genome assembly.

### Accession numbers

The raw sequence data is available under the accession number ERP000181 at the European Nucleotide Archive, ENA. The sequence and annotation data for *S.* Bovismorbificans strain 3114 chromosome and virulence plasmid, pVIRBov are available from ENA under accession numbers HF969015-HF969016.

## Results and Discussion

### Phylogenomics of *S.* Bovismorbificans isolates

To establish a phylogenetic framework for the *S.* Bovismorbificans samples we sequenced the genomes of 18 isolates, 14 of which were derived from Malawian adults and children isolated between 1997 and 2004 at the Queen Elizabeth Hospital Blantyre, Malawi. Suspecting these could be clonal, we brought into the analysis the sequences of 4 further isolates of different origin (pigs and alpaca), geographical location (UK) and temporal isolation (1970s/80s) to provide context to investigate wider serovar variations. Genomic DNA from the *S.* Bovismorbificans isolates were assayed using either 454 or multiplex Illumina sequencing (see methods). The genome data of these sequences are summarised in Table 1. Only chromosomal SNPs were used to construct the maximum likelihood phylogenetic tree (see methods) shown in Figure 1 (left), for which the root branch (leading to sample 51892776) was previously identified by including an outgroup (see methods). Recombinant regions and regions that were unlikely to reflect the core phylogeny, such as prophage, were removed from this analysis (48,423 total sites; Table S2).

**Table 1:** *S.* Bovismorbificans isolates used in this study.

Strain	Isolation year	Host	Age#	Outcome	pVIRBov	Antimicrobial Resistance profile	MLST ST*	ROD13	ROD14	ROD34	Accessory Genome Size (bp)
**human**											
3114	1997	child	ND	ND	+	RL_CXM_RD	142	+	+	+	0
3180	1997	child	ND	ND	+	RL_CXM_RD	142	+	+	+	38,420
3476	1997	child	ND	ND	+	RL_CXM_RD	142	+	+	+	131,557
D993	1999	child	ND	ND	+	CXM_RD	142SLV_1	+	+	+	110,972
D1253	1999	child	4M	ND	+	CXM_RD	142	+	+	+	5,577
D4451	2000	child	1Y11M	ND	+	RL_CXM_RD	142	+	+	+	4,791
D4891	2000	child	11M	ND	+	RL_CXM_RD	142SLV_2	+	+	+	4,791
A1104	1998	adult	ND	3	+	CXM_RD	142	+	+	+	4,791
A1608	1998	adult	ND	ND	+	RL_CXM_RD	142	+	+	−	7,893
A1668	1998	adult	ND	1	+	RL_CXM_RD	142	+	+	−	7,893
A16982	2002	adult	ND	2	+	RL_CXM_RD	142	+	+	+	4,735
A31126	2004	adult	23Y	1	+	RL_CXM_RD	142	+	+	+	34,498
A5893	1999	adult	35Y	ND	+	RL_CXM_RD	142	+	+	+	40,582
A8737	2000	adult	30Y	ND	+	RL_CXM_RD	142	+	+	+	5,695
**veterinary**											
499208	<1980	alpaca	N/A	N/A	−	AML_CN_W_RL_C_TET_CXM_RD_S	142	/	−	−	289,627
653308	<1980	pig	N/A	N/A	−	CXM_RD_S	142	/	−	−	88,266
276608	<1980	pig	N/A	N/A	−	TET_CXM_RD	142	/	−	−	121,149
51892776	<1980	pig	N/A	N/A	+	CXM_RD	142	−	−	−	36,589

The table summarizes properties of the isolates used in this study, including the presence or absence of ROD13, -14 and -34, the presence of the virulence plasmid pVIRBov and the size of the accessory genome in each of the addtional 17 Illumina-sequenced *S.* Bovismorbificans genomes obtained in comparison to strain 3114.

# M = months, Y = years ; outcome 1 = death, 2 = survived, 3 = unknown, ND = no data; N/A = not applicable; resistance profile = sulphamethoxazole (RL), cefuroxime (CXM), rifampicin (RD), amocixillin (AML), gentamicin (CN), trimethoprim (W), chloramphenicol (C), tetracycline (TET), streptomycin (S); ST refers to the MLST sequence type as determined by Illumina sequencing and sequencing of PCR amplicons,

* SLV = Single Locus Variant; ROD13/ = partial or different ROD present.

Amongst our samples, we found a total of 954 variable sites randomly distributed around the *S.* Bovismorbificans chromosome, which is approximately one SNP per 4,742 bp or just under 0.001% nucleotide divergence, within the core regions. It is evident that the human and animal isolates are intermixed. To give an idea of the level of nucleotide divergence within the core genome the animal isolate 51892776 closest to the root, was separated by 328 or 341 SNPs from the reference human isolate 3114 or most the divergent isolate shown in the tree, respectively. Consistent with this, we extracted the sequences of the MLST loci from the whole genome sequence data. All strains belonged to the major *S.* Bovismorbificans sequence type ST142 except two Malawian strains D993 and D4891 which were single locus variants (SLV) of ST142 (see Table 1) but were not found to be located on long branches on the tree.

It is evident from Figure 1 (left) that the human *S.* Bovismorbificans isolates taken in Malawi are phylogenetically extremely closely related, when compared to each other. This level of sequence divergence is comparable to the evolutionary distance between the *S.* Typhimurium lineages causing invasive disease in Africa [41] which form two distinct lineages (differentiated from each other by 455 SNPs), that are separated from the nearest gastroenteritis lineages by >700 SNPs [41]. However, when including the animal isolates from the UK it is apparent that this limited variation is a feature of *S.* Bovismorbificans as a serovar despite temporal, geographic and host differences. Also, in contrast to the *S.* Typhimurium lineages causing invasive disease, there was no clustering within the human samples with age or year of isolation.

Together these data suggested that the *S.* Bovismorbificans isolates causing invasive disease in Malawi, unlike *S.* Typhimurium, were not a specialised clade, at least according to the core phylogeny, therefore we looked at the accessory genome for clues to the observed differences in disease outcome for these isolates causing invasive disease in Malawi.

### Accessory genomic regions within *S.* Bovismorbificans ST142 isolates

In order to visualise the variation across *S.* Bovismorbificans isolates we constructed a pan-genome. To do this we concatenated the whole genome sequence of strain 3114 (chromosome and virulence plasmid) as well as the regions found to be variable present in one or more isolate, in a non-redundant manner (see methods). Figure 1 (right) shows the read coverage of all isolates included in this study mapped against the pan-genome pseudomolecule (see methods).

There are three main regions of difference (RODs) in chromosome of strain 3114, denoted ROD_13, ROD_14 and ROD_34. These are the only significant regions of difference in the core genome being either absent or partially present in the four veterinary strains and variably distributed in the human isolates (Table 1 and Figure 1). They are predicted to encode prophage; ROD_34 and ROD_13 are highly similar to the *S.* Typhimurium prophage elements Gifsy-1 and Gifsy-2, respectively, and have therefore been termed Gifsy-like (See Table S4). ROD14 represents a novel 46.4 kb prophage inserted into a spermidine/putrescine operon on the *S.* Bovismorbificans 3114 genome (See below; Table S4).

**Figure 2:**
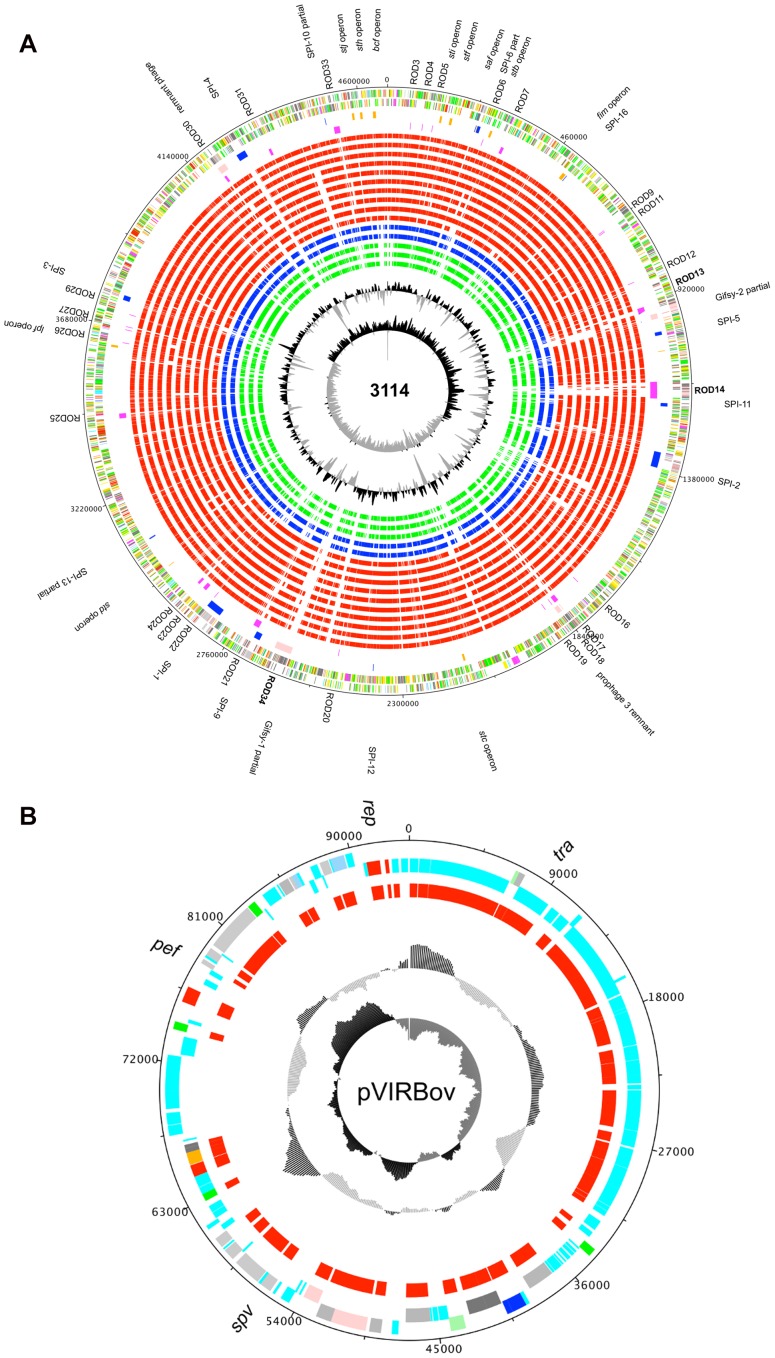
(A) Representation of the *S.* Bovismorbificans chromosome. From the outside in, the outer Circle 1 shows the size in base pairs. Circles 2 and 3 show the position of CDS transcribed in a clockwise and anti-clockwise direction, respectively. Circle 4 shows Regions of Difference (RODs) common to several NTS, including pathogenicity islands (blue), fimbrial operons (orange) and phages (pink), while Circle 5 shows (RODs) in *S.* Bovismorbificans that are different or absent from *S.* Typhimurium (magenta). Circles 6 to 20 show orthologous genes of *S.* Bovismorbificans (as determined by reciprocal FASTA analysis) in: *S.* Typhimurium (LT2), *S.* Typhimurium (D23580), *S.* Typhimurium (SL1344), *S.* Enteritidis (SEN), *S.* Cholaeraesuis (Schol), *S.* Paratyphi A (SpA), *S.* Paratyphi C (ParaC), *S.* Typhi (CT18), *S.* Gallinarum (SGAL) and *S.* Arizonae in red, *E. coli* (M1655) and *E. coli* (Sakai) in blue and *Yersinia enterocolitica* (YE), *Yersinia pestis* (YPSTB) and *Y. pestis* (YP91001) in green. Circle 21 shows a plot of G+C content (in a 10-kb window). Circle 22 shows a plot of GC skew ([G _ C]/[G+C]; in a 10-kb window). Genes in circles 3 and 4 are color-coded according to the function of their gene products: dark green, membrane or surface structures; yellow, central or intermediary metabolism; cyan, degradation of macromolecules; red, information transfer/cell division; cerise, degradation of small molecules; pale blue, regulators; salmon pink, pathogenicity or adaptation; black, energy metabolism; orange, conserved hypothetical; pale green, unknown; and brown, pseudogenes. **(B). The virulence plasmid of** ***S.*** **Bovismorbificans 3114 pVIRBov.** From the outside: Circle 1 shows the size in basepairs, Circle 2 and 3 show CDSs in a clockwise and anti-clockwise direction, respectively. Circle 4 shows othologous genes of pVIRBov in pSLT of *S.* Typhimurium LT2 (red) as determined by reciprocal fasta analysis. Circle 4 shows a plot of G+C content (in a 10-kb window). Circle 5 shows a plot of GC skew ([G _ C]/[G+C]; in a 10-kb window). Genes in circles 2 and 3 are colour-coded according to the function of their gene products: dark green, membrane or surface structures; cyan, degradation of macromolecules; red, information transfer/cell division; pale blue, regulators; salmon pink, pathogenicity or adaptation; black, energy metabolism; orange, conserved hypothetical; pale green, unknown.

A distinctive region of variation between human (carried in all) and animal samples (variably present) was a ∼93 kb virulence plasmid, here named pVIRBov (Figure 2(B)). pVIRBov is highly simlar to the *S.* Typhimurium LT2 virulence plasmid, pSLT (See Figure 2(B) and S2) and, like pSLT, carries the defining *spv* virulence gene cassette and the *pef* (plasmid-encoded fimbriae) operon mediating adhesion to murine intestinal epithelial cells [42]. Located downstream of the *pef* operon is the *rcK* (resistance to complement killing) gene. RcK is required by *S.* Typhimurium for survival in macrophages and for virulence in mice [43]. pVIRBov also shows a 7.463 kb deletion, and a 6.705 kb insertion, compared to pSLT. The deleted region contains a putative gene for single strand binding protein B (*ssbB*), as well as a number of putative genes encoding membrane-associated proteins. The insertion relative to pSLT includes a *pilA*-like gene (SBOV4711), *repC* (SBOV47701), a gene encoding a putative outer membrane protein (SBOV47871), *traJ* (SBOV48411), the primary activator of *tra* (polycistronic transfer) operon expression [44] and a number of hypothetical proteins. pVIRBov does not carry *rsk* (resistance to serum killing), thought to be associated with the control of serum resistance [45]. In contrast to a previous PCR based screen of *S.* Bovismorbificans isolates, these data show that 100% of the human bacteraeamia isolates and only one in four of the UK veterinary isolates carry pVIRBov [46].

In pairwise comparisons with respect to the *S.* Bovismorbificans str 3114 genome, eight human isolates (D1253, D4891, D4451, A1104, A1608, A1668, A16892 and A8737) were almost identical in genomic content to the reference, with accessory regions of only 4–8 kb (summarised in Table 1). The veterinary isolate 499208 and the paediatric bacteraemia isolate 3476 both carry the largest accessory genomic regions, of ∼290 kb and 132 kb, respectively (Table 1). The accessory genome of 3476 contains a ROD inserted at a t-RNA(Phe) -downstream of the CDS homologue to SBOV31771 in the 3114 strain-, showing high sequence homology to the SPI-7 of the human restricted pathogen *S.* Typhi CT18 (Figure 1, Figure S3). SPI-7 is a large mosaic pathogenicity island carrying a collection of virulence-related genes; versions of SPI-7 have been identified in *S. enterica* serovars Typhi, Paratyphi C, and Dublin [47]–[49]. This particular version of sample 3476 also encodes a putative Vi capsule although lacks the sopE phage of *S.* Typhi CT18. It also contains an operon related to carbohydrate metabolism and extracellular structure modifications (Figure S3). We did not find any genomic scar or any other evidence to show that this island might have been contained in any of the other human isolates or most recent ancestors.

Despite the existence of substantial accessory regions in half of the samples sequenced, they were mostly found to be unique to single isolates without correlation to the phylogenetic inference or the disease outcome.

### Comparative analysis of Pseudogene carriage amongst *S.* Bovismorbificans and *S.* Typhimurium isolates

In addition to gene gain, functional gene loss plays an important role in the adaptation of the *Salmonella* to different lifestyles, with host restricted *Salmonella* carrying over 200 pseudogenes [21], [22], [47], [50]–[52] compared to their broad host range counterparts. It is also evident that the patterns of gene loss are nonrandom with nonsense mutations and frame-shifts being over-represented in genes that are associated with aspects of virulence or host interaction. The parallels in the patterns of pseudogene accumulation is also a feature of the iNTS *S.* Typhimurium isolate D23580, causing invasive disease in Malawi.

By comparing the genome of strain 3114 to those of *S.* Typhimurium D23580, SL1344 and DT104, a total of 43 pseudogenes were identified in S. Bovismorbificans 3114. Six further *S.* Bovismorbificans isolates, which were chosen as representatives of distinct groups on the phylogenetic tree were also analysed for pseudogene content (summarised results in Table S5)

Of the 43 pseudogenes identified, 15 (34.9%) were conserved in all of the six *S.* Bovismorbificans isolates tested, but intact in the three *S.* Typhimurium strains. There was no clear distinction in pseudogene carriage between human bacteraemia (Malawi) and veterinary (UK) *S.* Bovismorbificans isolates, with only a single pseudogene specific to Malawian isolates. Five (11.5%) pseudogenes have been identified as pseudogenes in all seven *S.* Bovismorbificans and all three of the *S.* Typhimurium isolates (Table S5).

Genes that are pseudogenes in all seven *S.* Bovismorbificans isolates tested, but intact in all three *S.* Typhimurium isolates (D23580, SL1344, DT104), include genes involved in sugar metabolism, a putative autotransporter of the haemagglutinin family (SBOV37821) and putative surface-exposed virulence protein BigA (SBOV35501).

We observed no clear link between pseudogene carriage and source (human and veterinary isolates). Although the extent of pseudogene formation in *S.* Bovismorbificans does not compare to the host adapted *Salmonella* serovars or to the iNTS Typhimurium D23580, there are tantalising glimpses that suggest *S.* Bovismorbificans as a species may carry pseudogenes, such as BigA that are consistent with host specialisation. What is clear, though, is that this has not been driven by the emergence of a new dominant lineage adapted to humans in Africa as we have seen before with *S.* Typhimurium ST313.

### Detailed comparison of *S. Bovismorbificans* with other *S. enterica* genomes

**Figure 3:**
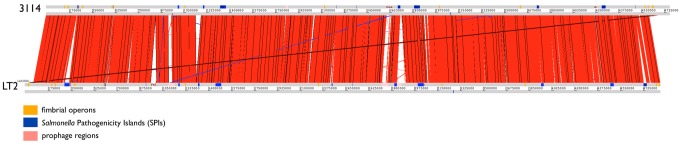
ACT comparison (http://www.sanger.ac.uk/Software/ACT) between *S.* Bovismorbificans 3114 and *S.* Typhimurium LT2. Showing amino acid matches between the complete six-frame translations (computed using TBLASTX) of the whole-genome sequences of *S.* Bovismorbificans and *S.* Typhimurium (LT2). Forward and reverse strands of DNA are shown for each genome (light grey horizontal bars). The red bars between the DNA lines represent individual TBLASTX matches, with inverted matches colored blue. The position of all the fimbrial operons marked in orange, the positions of *Salmonella* pathogenicity islands (SPI) are marked in blue, the position of prophages inserted into the genome are marked in pink. Analogous features are coloured the same.

Since this is the first time a *S.* Bovismorbificans genome has been described and to identify functions that may be involved in the apparent plastiticity in pathogenicity we performed whole-genome comparison between *S.* Bovismorbificans and *S.* Typhimurium LT2. These comparisons revealed a high level of synteny and colinearity, with no inversions (Figure 3). A comparison of *S.* Bovismorbificans genome statistics with other serovars is summarized in Table 2. Figure 2(A) shows orthologous genes, identified by reciprocal fasta searches, in 10 *S. enterica* subspecies 1 strains from eight *Salmonella* serovars as well as wider members of the *Enterobacteriaceae*. This analysis showed that *S.* Bovismorbificans gene content broadly resembles those common to *S. enterica* subspecies 1 NTS serovars. Genome comparisons to *S.* Typhimurium LT2 show that, while SPI-1,-2,-4,-5,-9 and -11 are largely synonymous, SPI-3,-6,-10 and -12 show deletions compared to the genome of *S.* Typhimurium LT2. Of note is SPI-6, formerly known as SCI (*Salmonella enterica* centisome 7 island), which is approximately 10.6 kb in size, compared to the 47 kb SPI-6 in the genome of *S.* Typhimurium LT2, and simply retains part of the fimbrial *saf* operon while lacking the Type VI secretion system (T6SS) encoded by this island. The T6SS is thought to play a role in adaptation to different lifestyles and environments, particularly animal hosts. SPI-6 T6SS was found to be absent from serovars Enteritidis, Gallinarum, Agona, Javiana, Virchow and IIIb 61∶1, v∶1,5,(7) [53]. Like *S.* Typhimurium LT2, SPI-13 and -14, are largely absent from *S.* Bovismorbificans [54] as are SPI-15 and -17 (Figure 3 see Table S6 for details on SPI repertoires)

**Table 2:** Properties of the *S.* Bovismorbificans 3114 chromosome compared to other *S.* enterica chromosomes [22], [47], [51], [72].

	Bovismorbificans	Typhimurium	Typhimurium	Typhi	Enteritidis	Gallinarum
strain	3114	D23850	LT2	CT18	P125109 (PT4)	287/91
Size (bp)	4677483	4879400	4857432	4809037	4685846	4658697
percent G&C	52.19	52.19	52.22	52.09	52.17	52.2
No of CDS	4671	4521	4451	4599	4318	4274
coding density	86.60%	86.30%	86.80%	87.60%	85.50%	79.90%
average gene size	877	947	947	958	953	939
pseudogenes	43	77	25	204	113	309

*S.* Bovismorbificans 3114 and *S.* Typhimurium LT2 share the same repertoire of 13 fimbrial operons (*stf, saf, stb, fim, stc, std, lpf, stj, sth, bcf, sti, csg* and *pef*) although *safA* of the *saf* operon is absent from the genome of strain 3114. The positions of the fimbrial operons within the *S.* Bovismorbificans 3114 genome are summarized in Figure 3.

### Comparison of *S. Bovismorbificans* with other *S. enterica* genomes: Regions of difference (RODs)

Regions of difference (RODs) were defined as insertions (or replacements) in the genome of *S.* Bovismorbificans 3114 when compared to published *S.* Typhimurium genomes (see methods; summarized in Table S4). A total of 27 RODs were identified many of which were predicted to encode proteins of unknown function. The most significant class of RODs were those related to prophage elements (ROD7,-12, -13, -14, -17, -21, -30, -31, 34; ROD 13, -14 and -34 are described above). Prophages are important sources of genomic variation in *Salmonella*, with most serovars being polylysogenic [55], [56]. Cryptic prophages have been shown to contribute to bacterial survival in adverse environments. They have been shown to help bacteria overcome acid, osmotic and oxidative stresses, influence growth and biofilm formation and contribute significantly to resistance to ß-lactams and quinolones [57].

In comparison to the genome of *S.* Typhimurium LT2, the genome of *S.* Bovismorbificans 3114 has a number of deletions or variations in regions related to common *Salmonella* prophages. There are no putative genes matching the common *Salmonella* prophage Fels-1 and Fels-2, with the exception of the Fels-1 *ybjP* gene. Also absent, compared to *S.* Typhimurium LT2, are inducible prophages Gifsy-1 and Gifsy-2, which have been replaced by prophage-like RODs 34 and 13 respectively (See above; Table S4). Although, ROD13 presents a partial match to Gifsy-2, unlike Gifsy-2, it does not carry the same genetic cargo *sodC1* which is associated with intracellular survival [58] or *gtgA*, which together with *sodCI* and *gtgB* is also absent from Gifsy-1 of *S.* Typhi [59]. ROD34 is 45.8 kb in size and carries Gifsy-1 like regions in both terminal regions, as well as one Fels-1 like region. ROD14 represents a novel prophage 46.4 kb in size with some similarities to a predicted *E. coli* (UMKN88) phage and to Bacteriophage P27. The cargo of ROD14 largely constitutes hypothetical proteins with the exception of the SPI-2 effector *sifA* gene (SBOV11471) which is essential for Sif formation, a process linked to SCV (*Salmonella* Containing Vacuole) integrity [60], [61]. The remaining prophage regions show no similarity to other *Salmonella* prophage but are present in all of the strains we have sequenced, regardless of source (summarised in Table S4)

### *S.* Bovismorbificans ST142 antibiotic resistance phenotypes and genotypes

Multidrug resistance is a serious problem in sub-Saharan Africa. *S.* Typhimurium and *S.* Enteritidis isolates from Malawi exhibit extensive resistance profiles. The empirical treatment for adults with sepsis in Malawi was chloramphenicol and benzyl penicillin. With the emergence of chloramphenicol resistance in *S.* Typhimurium isolates in 2002, treatment was switched to ciprofloxacin and parenteral gentamicin was added. While *S.* Typhimurium isolates have been resistant to ampicillin and trimethoprim-sulphamethoxazole for a long time, a dramatic increase of resistance to ampicillin, trimethoprim-sulphamethoxazole and chloramphenicol was observed in *S.* Enteritidis isolates in 1999 [62].

Phenotypic antibiotic resistance profiles (Table 1 and Figure 1) were obtained for all *S.* Bovismorbificans samples in this study. Of the 14 Malawian *S.* Bovismorbificans isolates, 11 showed resistance against sulphamethoxazole, cefuroxime and rifampicin, while three isolates were resistant to cefuroxime and rifampicin only. Contrary to the findings for *S.* Typhimurium and *S.* Enteritidis, *S.* Bovismorbificans isolates remain susceptible to chloramphenicol and ampicillin, and resistance to sulphamethoxazole and cefuroxime follows no dicernible temporal distribution.

Table 3 and Table S7 summarize putative resistance related genes identified in both the core and accessory genomic regions of all *S.* Bovismorbificans isolates. Consistent with other studies in enteric bacteria, linking antibiotic resistance phenotype and genotype was problematic [63]. However, we were able to identify a number of putative β-lactamase genes in *S.* Bovismorbificans that may explain the resistance to cephalosporins, such as Cefuroxime. Moreover all the *S.* Bovismorbificans isolates (including the veterinary ones) carry the mutation in the *rpoB* gene associated with resistance to rifampicin [64]–[66] (Figure S4 shows the *rpoB* gene alignment of strain 3114 together with those of *S.* Typhimurium LT2 and DT104). Despite phenotypic resistance to sulphamethoxazole no *sul* genes could be identified in any of the 14 Malawian *S.* Bovismorbificans isolates to explain this.

**Table 3:** Antimicrobial resistance genes identified in the *S.* Bovismorbificans genome.

Resistance gene	Mechanism of resistance	Resistance to*	SBOV CDS	DT104 CDS
*aadA*	streptomycin/spectinomycin adenyltransferase	SPT, STR	SBOV12521	SDT1236
*aphA*	aminoglycoside phosphotransferase	KAN	SBOV43311	SDT4236
*dhfr1*	dihydrofolate reductase	TMP	SBOV00351	SDT0092
putative β-lactamase	β-lactamases	PENs	SBOV25431	SDT2527
β-lactamase domain			SBOV29851	SDT2944
putative β-lactamase			SBOV38301	SDT3717

This table summarizes antimicrobial resistance genes identified in the S. Bovismorbificans core genome in comparison to the *S.* Typhiumurium DT104 genome.

* SPT = spectinomycin, STR = streptomycin,TMP = trimethoprim, KAN = kanamycin, PENs = penicillins.

Prevalence of tuberculosis (TB) is high in HIV-infected patients [67], and co-infection with invasive NTS and TB is common [68]. Rifampicin is the standard treatment for TB. Ours and previous data show that all of the NTS isolates, regardless of serovar, were resistant to rifampicin [11]. It is not possible to comment on whether co-infection and treatment for TB might be a causal mechanism for the emergence of resistance among *S.* Bovimorbificans or simply allowed an already resistant *S.* Bovimorbificans to exploit this niche. There is, however, strong evidence antimicrobial resistance, particularly to chloramphenicol in the case of *S.* Typhimurium have been key drivers in the spread of *Salmonella* pathovars across Africa [41].

A number of additional putative resistance genes were identified in the *S.* Bovismorbificans core genome, including those directed against aminoglycosides (*aadA*), streptomycin/spectinomycin (*aphA*) and trimethoprim (*dhfr1*) (Table S7).

For completeness the four veterinary UK isolates compared to the human isolates showed an even more diverse antimicrobial resistance profiles (summarised in Table 1), some of them showing an extensive array of resistance related genes, perhaps associated with the type of antibiotics and dosages used in the UK at the time they were isolated.

### Conclusion

In conclusion all *S.* Bovismorbificans isolates included in this study showed extremely close phylogenetic relationships regardless of source, place of isolation, host or disease outcome, even though morbidity and mortality caused by NTS is much more severe in sub-Saharan Africa and the developing world [22], [68]. Genome comparisons between the Malawian bacteraemia and UK veterinary isolates showed few clear differences. In our study, all of the bacteraemia isolates from Malawi were of the most prevalent *S.* Bovismorbificans sequence type, ST142.

Unlike iNTS *S.* Typhimurium isolates causing invasive disease in Malawi there is no evidence that functional gene loss was a significant feature of the evolution and adaptation to a more invasive lifestyle for African *S.* Bovismorbificans isolates. The only differences from those strains circulating elsewhere were in the mobile genome, largely prophage, and the presence of the virulence plasmid (only in one of four of the UK veterinary samples). However comparing the accessory genomic variations of the African *S.* Bovismorbificans isolates, such as the apparently random presence or absence of SPI-7, it strongly suggested that those causing disease originate from a mixed population of bacteria circulating within the region and that invasive disease by this serovar was caused by multiple sporadic independent bacteraemia infections.

All isolates, regardless of source, appear to display multiple phenotypic and genotypic drug resistance markers. In Malawi this is likely to have been essential to colonise a susceptible population, which tend to take regular antibiotic therapy. Although there is no obvious sign of convergent evolution to a more human adapted iNTS variant of *S.* Bovismorbificans, these strains -considering their importance in causing disease in this region-, may be at the very beginning of this process and so this study provides the reference point against which to compare changes that may become fixed in future lineages in sub-Saharan Africa. This study also highlights the likely importance of the patterns of evolutionary change we have previously highlighted in *S.* Typhimurium and show how, given the opportunity, multiple *Salmonella* serovars are able to cause more acute disease in susceptible populations.

## Supporting Information

Figure S1 **Concatenated MLST sequences of** ***S. enterica*** **subsp. I published on the MLST database (** www.mlst.net **).** Tip labels show major lineages of the *Salmonella enterica* subsp. I serovar identified (ST11-*S.* Enteritidis, ST15-*S.* Heidelberg, ST19-*S.* Typhimurium and ST142-*S.* Bovismorbificans. *S.* Typhi ST1 has been included as an outlier.
(TIF)

Figure S2 **ACT comparison (** http://www.sanger.ac.uk/Software/ACT **) between** ***S.*** **Bovismorbificans virulence plasmid pVIRBov (top) and** ***S.*** **Typhimurium LT2 virulence plasmid pSLT (AJ011572, bottom).** Showing amino acid matches between the complete six-frame translations (computed using TBLASTX) sequences of pVIRBov and pSLT. Forward and reverse strands of DNA are shown for each genome (light grey horizontal bars). The blue bars between the DNA lines represent individual TBLASTX matches, with inverted matches colored red. All genes present are colour-coded according to the function of their gene products: dark green, membrane or surface structures; cyan, degradation of macromolecules; red, information transfer/cell division; pale blue, regulators; salmon pink, pathogenicity or adaptation; black, energy metabolism; orange, conserved hypothetical; pale green, unknown. Analogous features are coloured the same.
(TIF)

Figure S3 **A SPI7 island on the accessory genome of** ***S.*** **Bovismorbificans 3476.** An EasyFig representation [69] showing comparison between the sequence of the reference *S* Bovismorbificans str 3114 (A) at the location where the SPI7 island of sample 3476 (B) is inserted on its own genome and with respect to S. Typhi CT18 (C). The new 97 kb SPI7 island (B) is most similar to that of S Typhi CT18 (C), containing an operon extra (genes in orange, marked B1) involved in carbohydrate modifications.
(TIF)

Figure S4 **ClustalW2 alignment of** ***rpoB*** **from** ***S.*** **Typhimurium LT2, DT104 and** ***S.*** **Bovismorbificans 3114.** Snapshot of ClustalW2 alignment [70], [71] of a section of the predicted amino acid sequences of *rpoB* from *S.* Typhimurium LT2, *S.* Typhimurium DT104 and *S.* Bovismorbificans 3114, highlighting the single amino acid change detected in 3114.
(TIFF)

Figure S5 **Putative function of CDS in** ***S.*** **Bovismorbificans accessory genomes according to blastx, measured in kilobases (kb) (** http://blast.ncbi.nlm.nih.gov/Blast.cgi **).**
(TIF)

Table S1 **Assembly data and statistics for Illumina sequenced genomes of** ***S.*** **Bovismorbificans isolates.**
(XLSX)

Table S2 **Nucleotide sites masked when reconstructing the phylogeny of** ***S.*** **Bovismorbificans isolates.**
(XLS)

Table S3 **SNP profile for all** ***S.*** **Bovismorbificans isolates sequenced in this study.**
(XLS)

Table S4 **Regions of difference (RODs) determined by ACT comparison of** ***S.*** **Bovismorbificans 3114 and** ***S.*** **Typhimurium LT2.**
(DOCX)

Table S5 **Summary of pseudogenes identified in strain 3114.**
(DOCX)

Table S6 **Comparison of** ***Salmonella*** **Pathogenicity Islands (SPI) repertoire of** ***S.*** **Bovismorbificans 3114 and** ***S.*** **Typhimurium LT2.**
(DOCX)

Table S7 **Resistance related genes identified in** ***S*** **Bovismorbificans samples.**
(XLSX)
