# Supplementary figures and images for: Genomic Characterisation of Invasive Non-Typhoidal Salmonella enterica Subspecies enterica Serovar Bovismorbificans Isolates from Malawi
Source: PLoS Negl Trop Dis. 2013 Nov 14;7(11):e2557. doi: 10.1371/journal.pntd.0002557 (PMC3828162; doi:10.1371/journal.pntd.0002557)

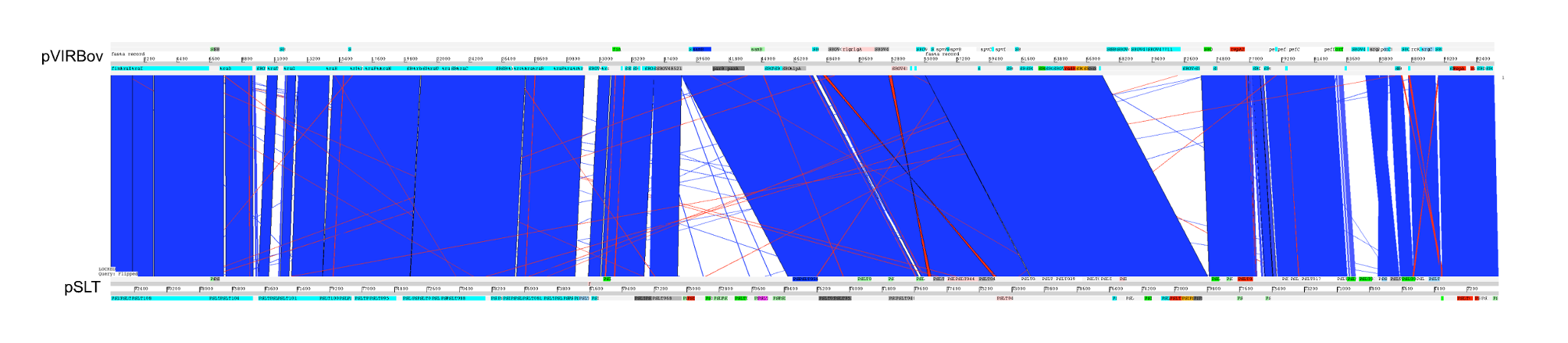

Supplement: Figure S2 — ACT comparison ( http://www.sanger.ac.uk/Software/ACT ) between S. Bovismorbificans virulence plasmid pVIRBov (top) and S. Typhimurium LT2 virulence plasmid pSLT (AJ011572, bottom). Showing amino acid matches between the complete six-frame translations (computed using TBLASTX) sequences of pVIRBov and pSLT. Forward and reverse strands of DNA are shown for each genome (light grey horizontal bars). The blue bars between the DNA lines represent individual TBLASTX matches, with inverted matches colored red. All genes present are colour-coded according to the function of their gene products: dark green, membrane or surface structures; cyan, degradation of macromolecules; red, information transfer/cell division; pale blue, regulators; salmon pink, pathogenicity or adaptation; black, energy metabolism; orange, conserved hypothetical; pale green, unknown. Analogous features are coloured the same. (TIF) [file pntd.0002557.s002.tif]

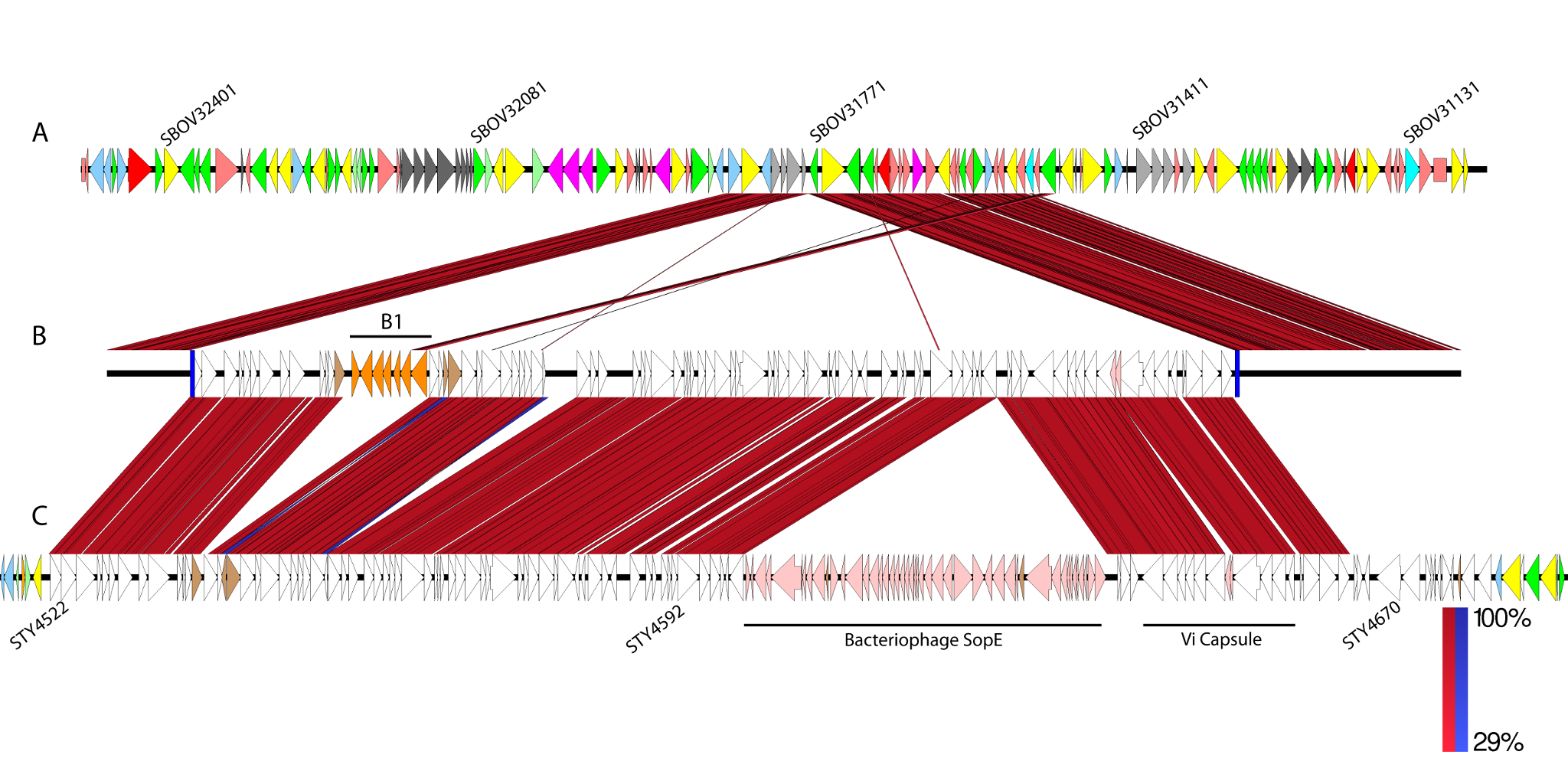

Supplement: Figure S3 — A SPI7 island on the accessory genome of S. Bovismorbificans 3476. An EasyFig representation [69] showing comparison between the sequence of the reference S Bovismorbificans str 3114 (A) at the location where the SPI7 island of sample 3476 (B) is inserted on its own genome and with respect to S. Typhi CT18 (C). The new 97 kb SPI7 island (B) is most similar to that of S Typhi CT18 (C), containing an operon extra (genes in orange, marked B1) involved in carbohydrate modifications. (TIF) [file pntd.0002557.s003.tif]

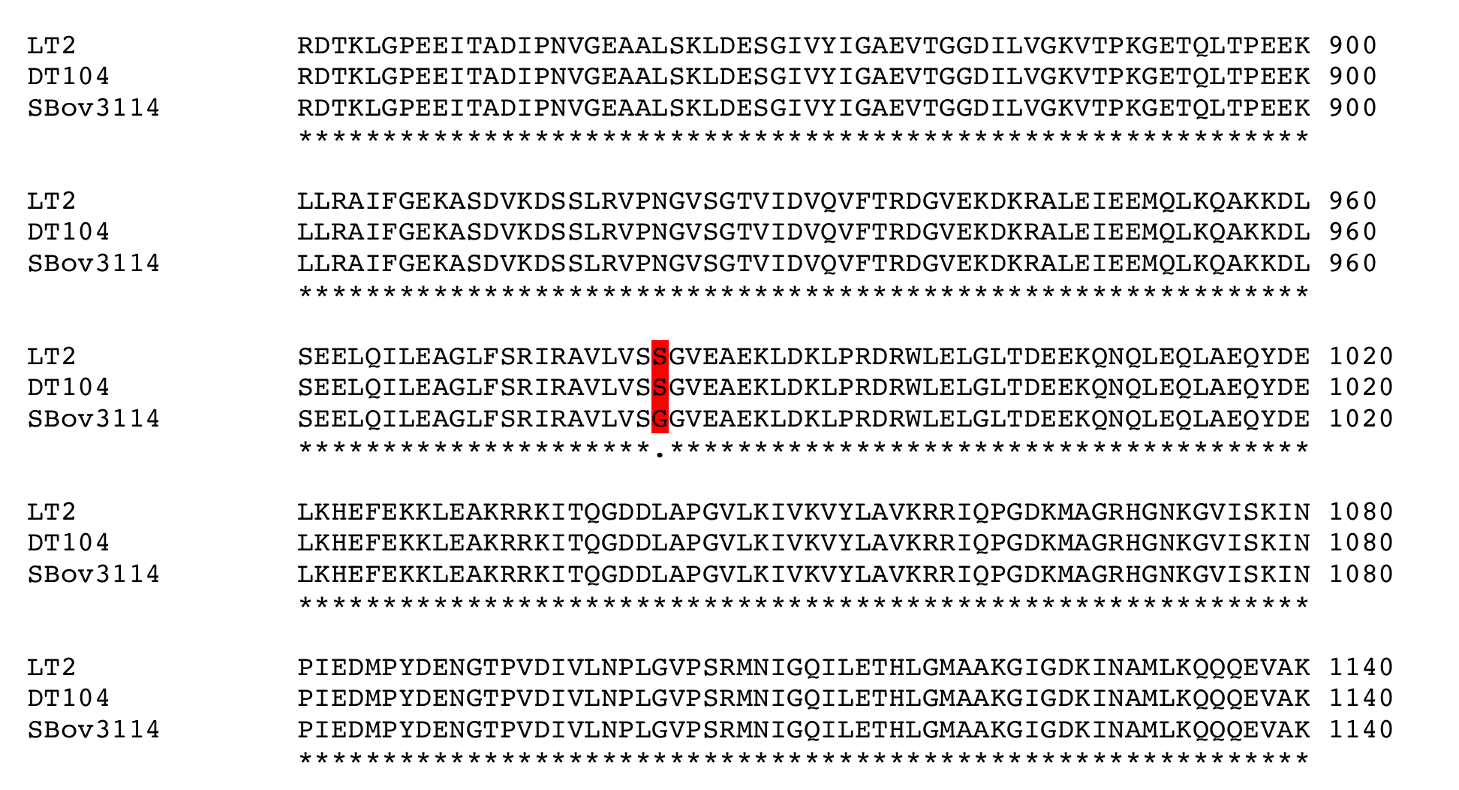

Supplement: Figure S4 — ClustalW2 alignment of rpoB from S. Typhimurium LT2, DT104 and S. Bovismorbificans 3114. Snapshot of ClustalW2 alignment [70], [71] of a section of the predicted amino acid sequences of rpoB from S. Typhimurium LT2, S. Typhimurium DT104 and S. Bovismorbificans 3114, highlighting the single amino acid change detected in 3114. (TIFF) [file pntd.0002557.s004.tiff]

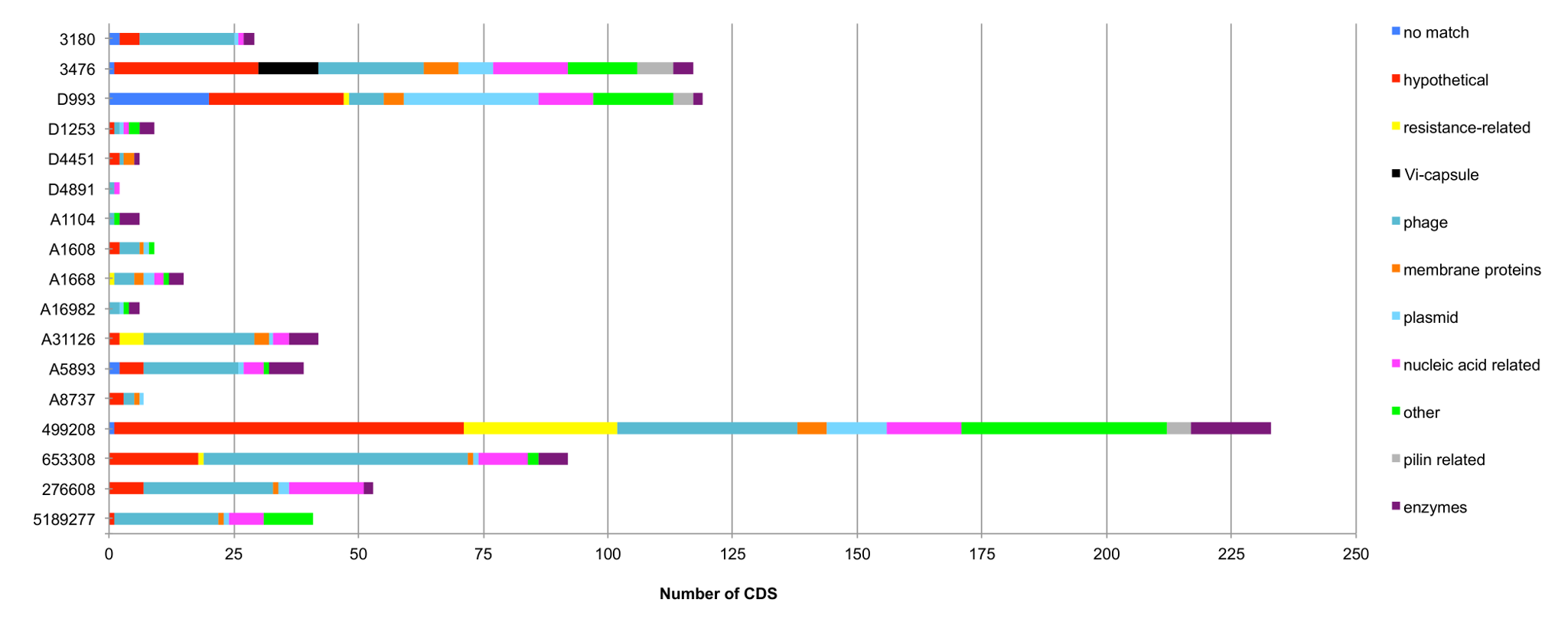

Supplement: Figure S5 — Putative function of CDS in S. Bovismorbificans accessory genomes according to blastx, measured in kilobases (kb) ( http://blast.ncbi.nlm.nih.gov/Blast.cgi ). (TIF) [file pntd.0002557.s005.tif]
