# Supplementary material for: Genomic Characterisation of Invasive Non-Typhoidal Salmonella enterica Subspecies enterica Serovar Bovismorbificans Isolates from Malawi
Source: PLoS Negl Trop Dis. 2013 Nov 14;7(11):e2557. doi: 10.1371/journal.pntd.0002557 (PMC3828162; doi:10.1371/journal.pntd.0002557)
Supplement: Table S4 — Regions of difference (RODs) determined by ACT comparison of S. Bovismorbificans 3114 and S. Typhimurium LT2. (DOCX) [file pntd.0002557.s009.docx]

**Table S4**. **Regions of difference (RODs) determined by ACT comparison of *S*. Bovismorbificans 3114 and *S*. Typhimurium LT2**

|  | ROD | label | CDS | Size of ROD (bp) | D23580 match and CDS | DT104 match and CDS | SL1344 match and CDS | putative function |
| --- | --- | --- | --- | --- | --- | --- | --- | --- |
| 1 | 3 | hypothetical | SBOV00591 | 498 | different | absent | absent | hypothetical |
| 2 | 4* | oxidoreductase | SBOV00901-SBOV00911 | 1755 | absent | absent | absent | aldo/keto reductase, transcriptional regulator LysR family |
| 3 | 5 | hypothetical | SBOV01161-SBOV01181 | 2020 | Different STM MW01651 | Different SDT0165 | partial match SL0160-SL0161 | hypothetical, phosphotyrosine protein phosphatase, yacL pseudo |
| 4 | 6 | Pathogenicity island-related | SBOV02291- SBOV02411 | 7911 | different STM_MW02641 – STM_MW03401 | partial match SPI-6 remnant | partial match SPI6 | hypothetical proteins, exported pathogenicity island protein transposase, putative cytoplasmic protein, Saf-pilin pilus formation protein, start of SPI-6 |
| 5 | 7 | prophage-related | SBOV02701, | 780 | different BTP1 | Different prophage 1 (ST104) | partial match SL0319-SL0321 | IS3 transposase orfA, pseudogene |
| 6 | 9 | membrane-related | SBOV05111-SBOV05121 | 3240 | different STM_MW06211-STM_MW06241 | Different SDT0580-SDT0584 | Different SL0546-SL0548 | putative membrane protein, glycosyl transferase |
| 7 | 11 | hypothetical | SBOV06871 | 705 | partial difference tRNAs | partial match tRNAs | partial match tRNAs | hypothetical |
| 8 | 12 | integrase | SBOV08536-SBOV08541 | 1269 | partial difference STM_MW09591-STM_MW09611 | Different SDT0917-SDT0919 | Different SL0885-SL0887 | hypothetical, phage integrase |
| 9 | 13 | Gifsy-2 related prophage | SBOV09131-SBOV09521 | 30159 | different BTP2 | different Gifsy-2 | different Gifsy2 | excisionase, exodeoxyribonuclease 8, putative regulator, diguanylate cyclase, alkyl/aryl-sulfatase BDS1, 27 hypothetical, phage holin-analog protein, bacteriophage lysis protein, phage terminase large subunit, Gifsy-1 prophage head-tail preconnector gp4 , gp5, phage major capsid protein E, Gifsy-1 prophage VmtH |
| 10 | 14* | remnant phage | SBOV11471 -SBOV12121 | 46413 | absent | absent | absent | SifA, 55 hypothetical, 2 exodeoxyribonuclease 8, replication P family, Eaa protein, HNH endonuclease, phage prohead protease, HK97 family, tail protein, spermidine/putrescine transporter, spermidine/putricine ABC transporter, putative peptidase T (aminotripeptidase) |
| 11 | 16 | hypothetical | SBOV18081-SBOV18091 | 2097 | absent | partially absent SDT1749-SDT1751 | partially absent SL1712-SL1713 | hypothetical |
| 12 | 17 | prophage remnant | SBOV18881-SBOV19071 | 12285 | partial match STM_MW18471-STM_MW18531 | partial match SDT1818-SDT1843 end of prophage 3 | partial match SL1785-SL1792 | putative cytoplasmic protein, 13 hypotehtical, pseudo, putative O-acetyl transferase related, putative phage integrase, transposase Mutator family, putative cytoplasmic, putative fimbriae usher |
| 13 | 18 | periplasmic proteins | SBOV19521-SBOV19551 | 3366 | partial match STM_MW18801 | partial match SDT1907-SDT1909 | absent | hypothetical, putative periplasmic protein, putative periplasmic phosphate binding protein |
| 14 | 19 | O-antigen related | SBOV21441-SBOV21551 | 11502 | Different STM_MW21141-STM_MW | different | different | phosphomannomutase, mannose-1-phospahte guanyltransferase/mannose-6-phosphate isomerase, glycosyl transferase group1, second mannosyl transferase, O-antigen polymerase, ramnosyl transferase, hypothetical, glycosyl transferase group 2 family protein, putative membrane protein, CDP-abequose synthase, |
| 15 | 20* | transcription | SBOV25581-SBOV25601 | 3915 | absent | absent | absent | glycerate kinase, gluconate transporter, transcriptional regulator CdaR |
| 16 | 34 | prophage-related | SBOV26591-SBOV27131 | 45774 | different BTP4 | different prophage 5 | partial match Gifsy-1 SLP272 | novel phage |
| 17 | 21 | prophage-related | SBOV27691-SBOV27871 | 16848 | different phage insertion | partial match, mostly different SDT2737-SDT2770 | partial match SL2712-SL2757 (region similar to CT18) | hypotheticals, phage DNA binding protein, phage immunity repressor protein, repressor of phase 1 flagellin gene, flagellin pseudo, H inversion protein |
| 18 | 22 | Cytoplasmic protein | SBOV29301-SBOV29351 | 3825 | partial match STM_MW28701 | absent | partial match SL2886-SL2887 | putative cytoplasmic protein, serine/threonine-protein phosphatase 2, hypotheticals |
| 19 | 23 | CRISPR sequences | SBOV29651- SBOV29791 | 10638 | partial match STM_MW29001-STM_MW29071 | mostly different SDT2931-SDT2938 | partial match CRISPR region-SL2923 | hypotheticals, CRISP-associated proteins Cas1, Cse3, Cas5, Cse4 family |
| 20 | 24 | fimbriae-related | SBOV29941- SBOV30001 | 6939 | absent/difference STM_MW29180 | absent | absent | putative fimbrial subunit, outer membrane usher protein, chaperone protein PapD, fimbrial subunit, putative minor fimbrial subunit, fimbrial subunit, hypothetical |
| 21 | 25 | membrane -related | SBOV35441- SBOV35501 | 13554 | match STM_MW34641-STM_MW34691 | match SDT3454-SDT3459 | match SL3440-SL3445 | putative membrane protein, hypothetical, nitrite reductase NAD(P)H small subunit, nitrite transporter NirC, siroheme synthase, porin autotransporter, |
| 22 | 26 | hypothetical | SBOV37321-SBOV37341 | 2070 | different | different | absent | hypothetical proteins, Putative transposase insK for insertion sequence |
| 23 | 27* | membrane-related | SBOV37701-SBOV37711 | 1917 | absent | absent | absent | secretion protein HlyD family protein, inner membrane protein YiaW, hypothetical |
| 24 | 29* | hypothetical | SBOV38131 | 980 | absent | absent | absent | hypothetical protein |
| 25 | 30 | prophage-related | SBOV42691-SBOV42741 | 6534 | different STM_MW41441-STM_MW41481 start of BTP6 | different SDT4181-SDT4186 (end of SPI) | partial match phage remnant SLP443 | DeoR family regulatory protein, hypothetical, putative membrane protein, tail assembly chaperone gp38, gp19 |
| 26 | 31 | prophage-related | SBOV44081- SBOV44161 | 7173 | partial match STM_MW42621-STM_MW42701 | partial match SDT4299-SDT4307 | partial match SL4248-SL4258 | araC family regulatory protein, cytoplasmic protein, hypothetical, putative periplasmic protein, TnpA pseudogene, putative acid phosphataseputative bacterial regulatory protein, merR cytochrome c-type biogenesis |
| 27 | 33 | membrane proteins | SBOV46131-SBOV46191 | 10422 | partial match STM_MW44641-STM_MW44761 | partial match SDT4501-SDT4514 | partial match SL4448-SL4461 | transporter, unknown function, endoribonuclease SymE, type I restriction-modification system – S and M subunit, type I restriction enzyme EcoEI R protein, putative membrane protein, |

* indicates RODs absent in all 3 *S*. Typhimurium (D23580, DT104 and SL1344)
