# Supplementary material for: Genomic Characterisation of Invasive Non-Typhoidal Salmonella enterica Subspecies enterica Serovar Bovismorbificans Isolates from Malawi
Source: PLoS Negl Trop Dis. 2013 Nov 14;7(11):e2557. doi: 10.1371/journal.pntd.0002557 (PMC3828162; doi:10.1371/journal.pntd.0002557)
Supplement: Table S5 — Summary of pseudogenes identified in strain 3114. (DOCX) [file pntd.0002557.s010.docx]

**Table S5** **Summary of pseudogenes identified in strain 3114**

|  | gene | strand | position | Putative function | Disabling mutation | **Human isolates Malawi (S. Bovismorbificans)** | | | | **Veterinary isolates UK (S. Bovismorbificans)** | | **Genome sequenced strains (S. Typhimurium)** | | |
| --- | --- | --- | --- | --- | --- | --- | --- | --- | --- | --- | --- | --- | --- | --- |
|  |  |  |  |  |  | **3180** | **D1253** | **D993** | **A1668** | **653308** | **276608** | **D23580** | **SL1344** | **DT104** |
| 1 | SBOV00231 | + | 86571-86756 | Putative uncharacterized protein | N-terminal missing | + | + | + | + | + | gap | var | absent | absent |
| 2 | SBOV02371 | + | 303720-304151 | exported pathogenicity island protein | pointmutation, premature stop codon | + | + | + | + | + * | + | var | absent | absent |
| 3 | SBOV02701 | - | 334555-334854 | IS3, transposase orfA | internal stop codon | gap | + | + | + | + | absent | - | - | absent |
| 4 | SBOV02711 | + | 335083-335362 | glycerol dehydratase reactivation factor large subunit | Large deletion, N-terminal missing, frameshift | + | + | + | + | + | absent | + | + | - |
| 5 | SBOV02791 | + | 341397-342361 | putative lysR family transcriptional regulator | frameshift | + | + | **-**** | **-**** | **-**** | **-**** | - | - | - |
| 6 | SBOV02961 | - | 357155-358613 | outer membrane protein OprM, truncated | deletion, frameshift | + | + | + | + | + | + | - | - | - |
| 7 | SBOV03101 | - | 377192-377353 | Transcriptional regulator, AraC family | partial | + | gap | + | + | + | absent | absent | absent | absent |
| 8 | SBOV03911 | + | 455204-456708 | Tetratricopeptide repeat protein | SFRP insertion. frameshift. | + | + | + | + | + | + | - | - | - |
| 9 | SBOV05141 | - | 581715-581960 | CopE1 | internal stop codon | + | + | + | + | + | + | absent | absent | absent |
| 10 | SBOV05171^$^ | + | 582601-582855 | cation efflux protein (fragment) | lacks start and stop | + | + | + | + | + | + | + | + | + |
| 11 | SBOV07771 | - | 850088-850531 | Sugar phosphatase SupH | 2 internal stop codons | + | + | + | + | + | + | - | - | - |
| 12 | SBOV08031 | + | 876031-876721 | glucose sorbosone dehydrogenase | 2 frameshifts, changes, | + | + | + | + | + | + | - | - | - |
| 13 | SBOV09641 | + | 1041774-1042772 | type III secreted protein | insertion of RSGFLPATIS | gap | gap | - | gap | gap | gap | + | - | - |
| 14 | SBOV15781^$^ | - | 1592832-1595879 | Putative molybdopterinoxidoreductase | internal stop codon | + | + | + | + | + | + | + | + | + |
| 15 | SBOV19011 | + | 1904739-1905454 | Lipoploysaccharide 1,2-N-acetylglucosamonetransferase | frameshift | + | + | + | + | + | gap | absent | absent | - |
| 16 | SBOV19141 | - | 1913248-1913469 | transposase | N-terminal missing | + | + | + | + | + | gap | absent | absent | absent |
| 17 | SBOV20031 | - | 1986395-1986726 | Putative inner membrane protein | frameshift | - | - | - | - | -** | absent | - | - | - |
| 18 | SBOV23111 | - | 2290990-2292035 | Cytochrome c-type biogenesis pro | frameshift | gap | absent | absent | + | + | gap | - | - | - |
| 19 | SBOV25041 | + | 2494928-2495467 | Putative membrane carboxypeptidase | internal stop codon | + | + | + | + | + | + | - | - | - |
| 20 | SBOV25051 | - | 2495373-2495552 | Putative carboxypeptidase | partial | + | + | + | + | + | + | absent | absent | - |
| 21 | SBOV25781 | - | 2573382-2579067 | AIDA autotransporter-like protein, SdhA | frameshift, indels | + | + | + | + | + | + | - | - | - |
| 22 | SBOV25791^$^ | - | 2579768-2587075 | putative outer membrane protein (RatB) | pointmutation in the start codon | + | + | + | + | + | + | + | + | + |
| 23 | SBOV26011 | - | 2619092-2619316 | Hypothetical protein | pointmutation at start codon | + | + | + | + | + | + | absent | absent | absent |
| 24 | SBOV27071 | + | 2713689-2713805 | putative phage protein (pseudogene) | partial | + | + | + | absent | + | gap | + | + | + |
| 25 | SBOV27101 | + | 2714109-2715710 | Gifsy-1 prophage RecE | partial | + | + | + | gap | absent | + | - | - | - |
| 26 | SBOV27341 | - | 2736284-2736738 | Putative cytoplasmic protein | frameshift | + | + | + | + | gap | gap | - | - | - |
| 27 | SBOV27861 | - | 2791881-2793542 | Phase 2 flagellin (*fljB*) | N-terminal missing | + | +* | +* | gap | gap | gap | - | - | - |
| 28 | SBOV29771 | - | 2962962-2964497 | Crispr-associated protein, Cse1 | internal stop codon | + | + | + | + | + | + | - | absent | absent |
| 29 | SBOV30701 | - | 3065265-3065435 | Putative uncharacterized protein | pointmutation in nt10, change in start codon | + | + | + | + | + | + | absent | absent | absent |
| 30 | SBOV34166 | - | 3400260-3401604 | Oxaloactetate decarboxylase, subunit beta | frameshift | gap | gap | gap | +* | del | gap | - | - | - |
| 31 | SBOV34721 |  | 3452444-3453567 | DprA (DNA protecting protein) | frameshift | + | + | + | - | + | + | - | - | - |
| 32 | SBOV35121 | - | 3475064-3476311 | Translation elongation factor EF Tu | frameshift | + | gap | absent | gap | gap | gap | - | - | - |
| 33 | SBOV35501 | + | 3505918-3511628 | Putative surface-exposed virulence protein BigA | frameshift, deletions | + | + | + | + | + * | + | - | - | - |
| 34 | SBOV37341 | - | 3709637-3710101 | Integrase/transposase | Internal stop codon | + | + | + | + | + | + | absent | absent | absent |
| 35 | SBOV37821 | + | 3753385-3757850 | Putative autotransporter, Haemagglutinin family | frameshift | + | + | + | + | + | + | - | - | - |
| 36 | SBOV39151 | - | 3880609-3881049 | Putative autotransporter | partial | absent | + | - | + | + | gap | - | - | - |
| 37 | SBOV39976 | + | 3972439-3972872 | Hypothetical protein | frameshift | + | + | + | + | + | gap | + | absent | + |
| 38 | SBOV40231 | - | 4003643-4004164 | Possible exported protein | 2 frameshifts | + | + | + | + | + | + | - | - | + |
| 39 | SBOV41941 | + | 4164170-4165726 | 5'-Nucleotidase domain protein | internal stop codon | + | - | + | - | + | - | - | - | - |
| 40 | SBOV43471 | + | 4320438-4335990 | large repetitive protein, putative inner membrane protein | frameshift | + | + | + | + | + | + | - | - | - |
| 41 | SBOV43741 | - | 4360965-4361126 | Putative uncharacterized pro | no close blast match | + | + | + | + | + | gap | absent | absent | absent |
| 42 | SBOV43781^$^ | - | 4362113-4364260 | Formate dehydrogenase H | internal stop codon | + | + | + | + | + | + | + | + | + |
| 43 | SBOV44141^$^ | - | 4400720-4401765 | TnpA transposase | 2 frameshifts, insertions and rearrangements | + | + | + | + | + | + | + | + | + |
|  |  |  |  |  |  |  |  |  |  |  |  |  |  |  |

+ indicates that the gene is a pseudogene; - indicates that the gene is intact (not a pseudogene);

“absent” indicates that the gene was absent from the genome;* indicates that there is a gap in the sequence but a mutation could still be identified in the remainder of the sequence;** indicates that there is a gap in the sequence but no frameshift was present;

“gap” indicates that there is a gap in the sequence;“del” there is a deletion in the genome compared to 3114;

“var” variation in the genome compared to 3114;$ pseudogene was present in all isolates tested
