# Supplementary material for: Genomic Characterisation of Invasive Non-Typhoidal Salmonella enterica Subspecies enterica Serovar Bovismorbificans Isolates from Malawi
Source: PLoS Negl Trop Dis. 2013 Nov 14;7(11):e2557. doi: 10.1371/journal.pntd.0002557 (PMC3828162; doi:10.1371/journal.pntd.0002557)
Supplement: Table S6 — Comparison of Salmonella Pathogenicity Islands (SPI) repertoire of S. Bovismorbificans 3114 and S. Typhimurium LT2. (DOCX) [file pntd.0002557.s011.docx]

**Table S6 Comparison of *Salmonella* Pathogenicity Islands (SPI) repertoire of *S*. Bovismorbificans 3114 and *S*. Typhimurium LT2** (size of SPIs in kb)

|  | SPI-1 | SPI-2 | SPI-3 | SPI-4 | SPI-5 | SPI-6 | SPI-7 | SPI-8 | SPI-9 | SPI-10 | SPI-11 | SPI-12 | SPI-13 | SPI-14 | SPI-15 | SPI-16 | SPI-17 |
| --- | --- | --- | --- | --- | --- | --- | --- | --- | --- | --- | --- | --- | --- | --- | --- | --- | --- |
| S Typhimurium LT2 | 42.04 | 41.72 | 16.52 | 24.96 | 9.05 | 46.69 | - | - | 16.91 | 21.10 | 9.03 | 4.70 | 0.4 partial  0.3 partial | 0.5 partial | - | 4.5 | - |
| S. Bovismorbificans 3114 | 42.04 | 41.74 | 12.95 | 25.47 | 9.05 | 10.63 | - | - | 16.92 | 0.33 | 8.94 | 1.01 | 0.4 partial  0.3 partial | 0.5 partial | - | 1.2 | - |
